# Understanding the relationship between pore size, surface charge density, and Cu^2+^ adsorption in mesoporous silica

**DOI:** 10.1038/s41598-024-64337-5

**Published:** 2024-06-12

**Authors:** Yanhui Niu, Wenbin Yu, Shuguang Yang, Quan Wan

**Affiliations:** 1 School of Chemistry and Materials Science, Guizhou Education University, Guiyang, 550018 China; 2 State Key Laboratory of Ore Deposit Geochemistry, Chinese Academy of Sciences, Institute of Geochemistry, Guiyang, 550081 China

**Keywords:** Mesoporous silica, Pore size, Surface charge density, Cu^2+^ adsorption, Materials chemistry, Structural properties, Geochemistry, Environmental chemistry

## Abstract

This research delved into the influence of mesoporous silica’s surface charge density on the adsorption of Cu^2+^. The synthesis of mesoporous silica employed the hydrothermal method, with pore size controlled by varying the length of trimethylammonium bromide (C_n_TAB, n = 12, 14, 16) chains. Gas adsorption techniques and transmission electron microscopy characterized the mesoporous silica structure. Surface charge densities of the mesoporous silica were determined through potentiometric titration, while surface hydroxyl densities were assessed using the thermogravimetric method. Subsequently, batch adsorption experiments were conducted to study the adsorption of Cu^2+^ in mesoporous silica, and the process was comprehensively analyzed using Atomic absorption spectrometry (AAS), Fourier transform infrared (FTIR), and L3 edge X-ray absorption near edge structure (XANES). The research findings suggest a positive correlation between the pore size of mesoporous silica, its surface charge density, and the adsorption capacity for Cu^2+^. More specifically, as the pore size increases within the 3–4.1 nm range, the surface charge density and the adsorption capacity for Cu^2+^ also increase. Our findings provide valuable insights into the relationship between the physicochemical properties of mesoporous silica and the adsorption behavior of Cu^2+^, offering potential applications in areas such as environmental remediation and catalysis.

## Introduction

Nanogeoscience is an emerging interdisciplinary field resulting from integrating Nanotechnology and Earth sciences, aiming to explore nanomaterials across various Earth strata and understand the nexus between nanoscale phenomena and geological processes shaping Earth’s evolution^1^. One key focus is the exploration of mineral nanopores^2^ classified by IUPAC as macropores (> 50 nm), mesopores (2–50 nm), and micropores (< 2 nm) to deepen the comprehension of Earth’s dynamic evolution through unraveling connections between nanoscale effects and geological manifestations.

Mineral mesoporous materials are ubiquitous in geological settings and play a significant role in Earth science phenomena. Minerals such as diatomaceous earth and halloysite exhibit abundant mesopores in their structures^3,4^, endowing them with pronounced selective adsorption capabilities. Hochella and Banfield^5^ have emphasized the significant influence of water’s intrinsic properties, such as high viscosity and low dielectric constant when confined within nanoscale pores. This influence affects the entire weathering process. Mesoporous structures in shale and tight sandstone are integral features of oil and gas reservoirs^6^. Furthermore, mineral mesopores are instrumental in addressing critical Earth science challenges such as carbon cycling^7^, mineral dissolution and precipitation^8^, and trace element enrichment^9^. This underscores the pivotal role of mineral mesopore adsorption behaviors as the central research focus in nanogeoscience, shaping various Earth science processes, including carbon cycling, mineral dissolution, and trace element enrichment mechanisms driven by their mesoporous architectures.

Heavy metal pollution is a significant environmental concern, with adsorption playing a crucial role in the fate of heavy metals in geological environments^10^. Mesoporous minerals with large specific surface areas exhibit effective heavy metal ions adsorption capabilities. Studying adsorption mechanisms onto mineral mesopores can enhance our understanding and prediction of heavy metal behavior in geological settings, offering insights into controlling and remediating heavy metal pollution and addressing scientific challenges related to trace elements and metal mineralization^11,12^.

Recent research has uncovered mineral mesoporous oxides’ superior heavy metal adsorption capacity compared to non-porous oxides^13,14^, highlighting the importance of investigating the nanoconfinement effect and surface charge density on heavy metal adsorption mechanisms. While some literature has touched on the effect of nanoconfinement in mesoporous silica, the detailed mechanisms of heavy metal ion adsorption still need to be fully understood and require further clarification^15,16^. The current literature needs more systematic research on the effect of nanopore size on the adsorption behavior of heavy metal ion ions, leading to an incomplete understanding of the underlying adsorption mechanisms and factors at play. More information is needed on how precise control of nanopore size can influence the adsorption behavior of heavy metal ions^17^, pointing to a need for more targeted research in this area. Furthermore, previous research needs to adequately emphasize the significance of surface charge density in the adsorption of heavy metal ions onto mineral mesopores. This oversight highlights an urgent need for further studies to investigate its impact on heavy metal adsorption more thoroughly. Surface charge density, which plays a pivotal role in the adsorption characteristics of substances and their interactions with ions and molecules, must be examined more in the existing literature^18^. The lack of quantitative control and a comprehensive understanding of surface chemical properties highlights a significant gap in our knowledge concerning the fundamental mechanisms driving technological advancements, particularly in practical application scenarios. Although synthetic mesoporous materials like mesoporous silica are leveraged as model systems to simulate complex natural compositions that affect adsorption in mineral nanopores^19^, these models must be refined to reflect real-world complexities more accurately. Doing so requires enhancing the quantitative control of their surface chemical properties and deepening our understanding of how these properties modulate heavy metal adsorption processes. By addressing these shortcomings, we can develop more effective and realistic models that provide valuable insights into the fundamental mechanisms of adsorption, thereby facilitating the advancement of practical applications in this field. Exploring surface charge density is essential for a thorough comprehension of the adsorption mechanisms of heavy metals, as it directly influences the charge properties on the mineral surface, the adsorption affinity of heavy metal ions, and the dissociation equilibrium on the mineral surface.

Building upon the comprehensive study of heavy metal ion sequestration in engineered mesoporous materials, we mainly focus on the scenario of Cu^2+^, a ubiquitous and ecologically consequential type of heavy metal ions. The detailed investigation of Cu^2+^ adsorption on mineral surfaces within mesoporous silica is a complex yet crucial aspect of nanogeoscience research, given the significant environmental impact of Cu^2+^. By utilizing a combination of sophisticated analytical techniques such as TEM, BET analysis, XRD, and spectroscopic methods, this research aims to unravel the intricate mechanisms governing the adsorption behavior of Cu^2+^ in mesoporous silica, with the ultimate goal of developing more efficient methods for heavy metal ion sequestration. Through a systematic exploration of pore size variations, surface charge density effects, and adsorption capacity, this study will contribute valuable insights into the reactivity of mineral interfaces at the nanoscale level and inform future efforts in environmental remediation.

In conclusion, this research endeavors to comprehensively understand the relationships between pore size, surface charge density, and Cu^2+^ adsorption in mesoporous silica. By shedding light on the underlying interactions and processes at play, this study not only enhances the scientific knowledge of heavy metal adsorption in geological contexts but also paves the way for future exploration in nanogeoscience research. Ultimately, the findings of this study will offer a nuanced perspective on the adsorption mechanisms within mesoporous silica and their implications in the broader field of environmental and material sciences.

## Results and discussion

### Structural characterization analysis of mesoporous silica (MPS)

**Figure 1:**
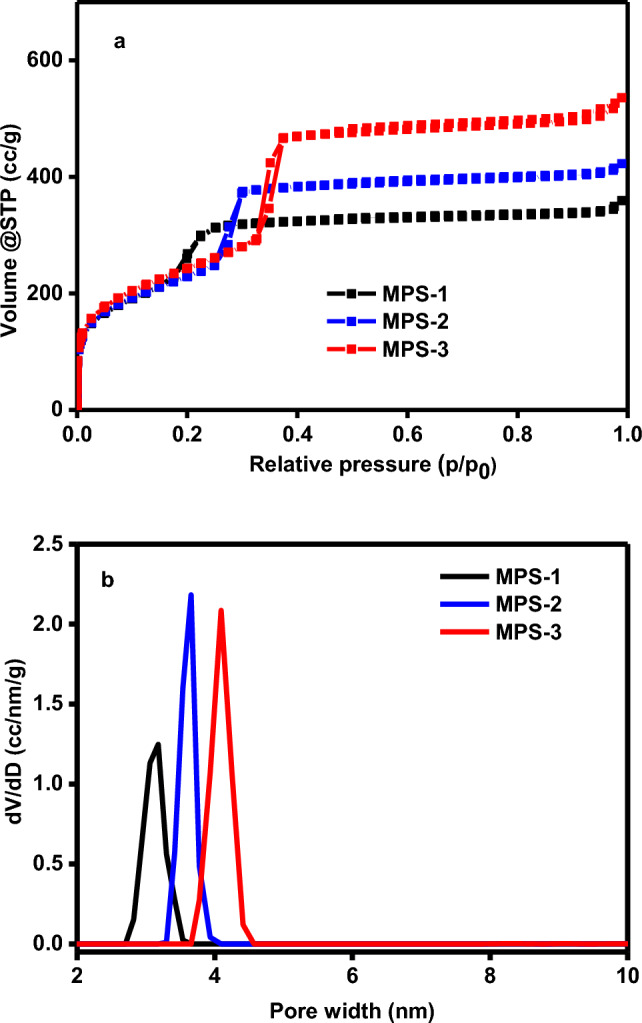
(**a**) N_2_ adsorption/desorption isotherm and (**b**) pore size distribution of MPS/.

**Figure 2:**
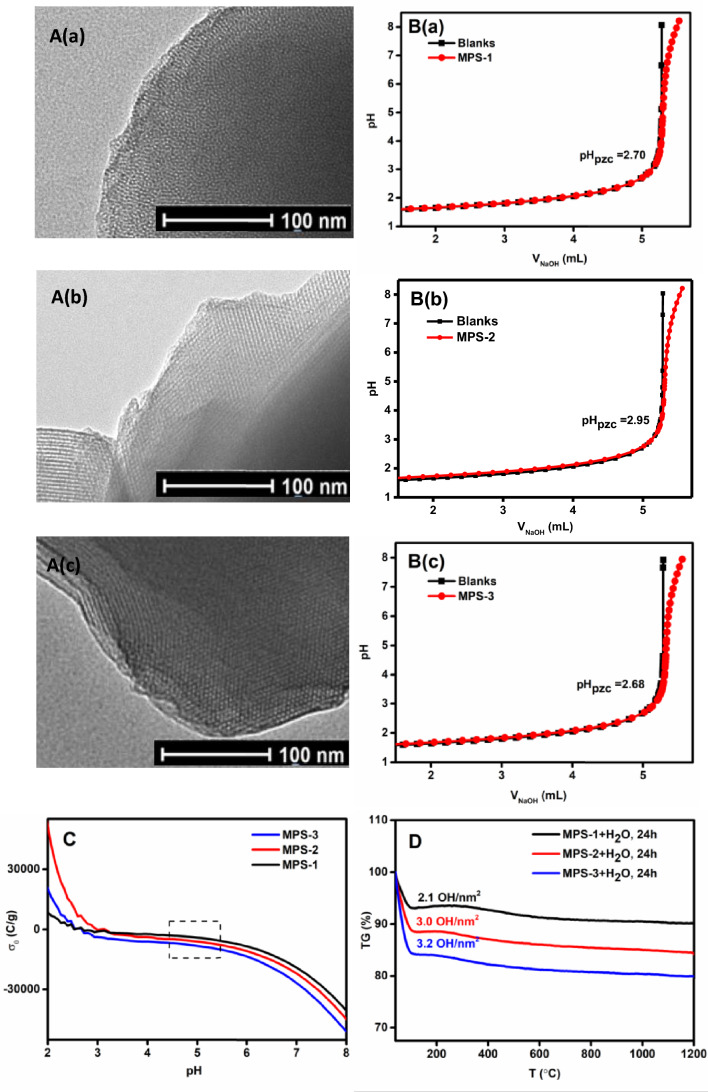
TEM images of MPS (**A**), Acid–base titration curve of MPS (**B**),The change curves of Surface charge with pH of MPS (**C**) and TG curves of MPS (**D**).

The N_2_ adsorption method is an effective way to examine mesoporous materials. In Fig. 1a, we can see the nitrogen adsorption/desorption isotherm of MPS at 77 K. According to the International Union of Pure and Applied Chemistry (IUPAC) classification, the plot matches a type IV adsorption isotherm, which is typical of mesoporous structures^20^. The plot shows that capillary condensation causes a sharp increase in adsorption. At low pressures, the adsorption grows linearly, indicating the monolayer adsorption of N_2_ on the pore walls. The steeper change in adsorption during the capillary condensation region suggests a more uniform pore size distribution. After capillary condensation, a long and stable plateau is reached at higher pressures, indicating the multilayer adsorption on the pore surface. Furthermore, the specimen with a pore size of 4.1 nm demonstrated higher adsorption than those with pore sizes of 3.2 and 3.7 nm. This means that the pore size influences the adsorption of N_2_ according to physical adsorption. However, the variation in chemical adsorption was not consistent with pore size changes because chemical adsorption generally occurs at chemically active sites^21^. The large specific surface area and uniform pore size distribution (as shown in Fig. 1b) of the two-dimensional hexagonal mesoporous structure also agreed with the TEM (as shown in Fig. 2A) and XRD results(as shown in Figure S1).Therefore, these MPS samples with different pore sizes were further selected for Cu^2+^ adsorption experiments.

### Analysis of the relationship between pore size and surface charge density

Amphoteric oxides carry positive charges at low pH levels and negative charges at high pH levels^22^. This allows us to determine the solid amphoteric oxide’s point of zero charge (pH_PZC_). In this study, we used the titration method proposed by Kosmulski^23^ to obtain the titration curves of the blank solution and the sample suspension. By calculating the difference between the acid and base titration curves at the same pH, we determined the surface charge density σ_0_ using the formula: σ_0_ = F c∆V/(mA) (where, σ_0_ is in C/m^2^, F is the Faraday’s constant (96487 C/mol), c is the molar concentration of the added base (mol/L), m is the solid quality, and A is the solid specific surface area in m^2^/g). The intersection of these two titration curves is considered the apparent PZC. The titration and surface charge curves are shown in Fig. 2B and C, respectively. The apparent pH PZC was found to be between 2.6–3. The surface charge densities calculated for MPS-1, MPS-2, and MPS-3 are listed in Table 1. A noticeable increasing trend is observed as the pore size expands. This trend is attributed to the combined effect of the pore size on the charge regulation in silica nanopores, while also considering that the overlapping of electrical double layers (EDL) becomes negligible^23^.

**Table 1:** Structural characteristic parameter of the MPS samples.

Sample	S_BET_ (m^2^/g)	D_DFT_ (nm)	V_DFT_ (cc/g)	Si–OH (/nm^2^)	σ_0_ (10^−2^ C/m^2^)	σ_0_ (e^−^/nm^2^)	Exp.Q_e_ (Cu^2+^, mg/g)
MPS-1	842	3.2	0.5	2.1	0.5	0.03	0.09
MPS-2	840	3.7	0.6	3	0.7	0.044	0.15
MPS-3	882	4.1	0.8	3.2	0.9	0.057	0.23

The surface charge density of silica is usually controlled by protonation and deprotonation reactions, which belong to the category of solid surface ionization. These reactions are influenced by counterions and are referred to as “charge screening,” which implies that co-ions accumulate and counter ions are repelled^24^. Normally, the interfacial region is electrically neutral. However, it maintains its electrical neutrality under the influence of counter ions from the surface layer (the inert electrolyte) when protons are adsorbed or dissociated. The surface charge of mesoporous silica is directly related to the surface acidity/basicity ratio, namely the pH level and ionic strength, and can be evaluated using acid–base titration methods.

When a solid surface is charged in a solvent, according to electroneutrality, an equal amount of the opposite charge should exist on the other side of the surface, namely the solvent side. This forms what is known as a double layer^25^. In the case of silica, the ionization in water can provide information on its surface charge properties in the solution. At pH 5, the pore walls of mesoporous silica (MPS) carry negative charges through the dissociation of silanol groups. This generated potential attracts positively charged counterions from the aqueous solution, making the entire system electrically neutral and forming the double layer. The Debye layer, which is the negative charge layer, combines with the pore wall surface. Its primary function is to generate negative charges on the pore walls, thus increasing their potential. When the thickness of the Debye layer is larger than the mesopore radius, the potential to the pore wall and the double layer overlap, causing the central potential of the mesopores to no longer be zero. Silica microchannels with hydroxyl groups have an isoelectric point of 2–3, and in solutions with a pH higher than 3, the silica surface carries negative charges. When the diameter of MPS is at the nanoscale (2–5 nm), the surface double layer may overlap. In summary, the charge density on the solid surface of MPS is related to the pore size, surface functional groups, pH of the solution, and ionic strength. With quantitative data on mesoporous silica’s surface charge and hydroxyl density^26^, one can explain the mechanism of heavy metal adsorption on mesoporous materials at a certain pH level.

In Fig. 2D, we can see the TGA curve that was obtained after mixing MPS with deionized water for 24 h. Based on various reports^23^, the dehydration process of mesoporous silica can be described as follows: the weight loss before 200 °C is because of the desorption of physically adsorbed water. On the other hand, the weight loss between 200 and 1200 °C is caused by the dehydroxylation of silicon hydroxyl groups. Therefore, we can calculate the density of silicon hydroxyl groups using a formula: $\delta_{OH} (Si - OH/nm^{2} ) = \frac{{\Delta m_{{H_{2} O}} }}{{18S_{BET} }} \times 2 \times 6.02 \times 10^{3}$. The silicon hydroxyl densities of MPS-1, MPS-2, and MPS-3 are presented in Table 1. The TGA curve shows a distinct weight loss starting at 200 °C, indicating an increase in the silicon hydroxyl density with increasing pore size. Although this method does not involve measurements in an in-situ adsorption system at pH 5, it indirectly explains the variation in surface hydroxyl contents of MPS with different pore sizes. This discrepancy can be attributed to the fact that a portion of the solution remains trapped within the mesopores and cannot be eliminated from the system.

### Analysis of the relationship between pore size and copper ion adsorption

#### The thermodynamics and kinetics of Cu^2+^ adsorption in MPS

Before determining the optimal conditions for the adsorption studies, a series of preliminary experiments were conducted to assess the influence of pH and the solids-to-liquid ratio on the adsorption process (as shown in Figure S2, S3). These preliminary tests were crucial in establishing the most favorable conditions for Cu^2+^ adsorption onto MPS. Based on the outcomes of these tests, a pH of 5 and a solids-to-liquid ratio of 5 g/L were identified as the optimal conditions. Subsequently, all adsorption experiments for the different pore sizes of MPS were carried out under these selected conditions.

**Figure 3:**
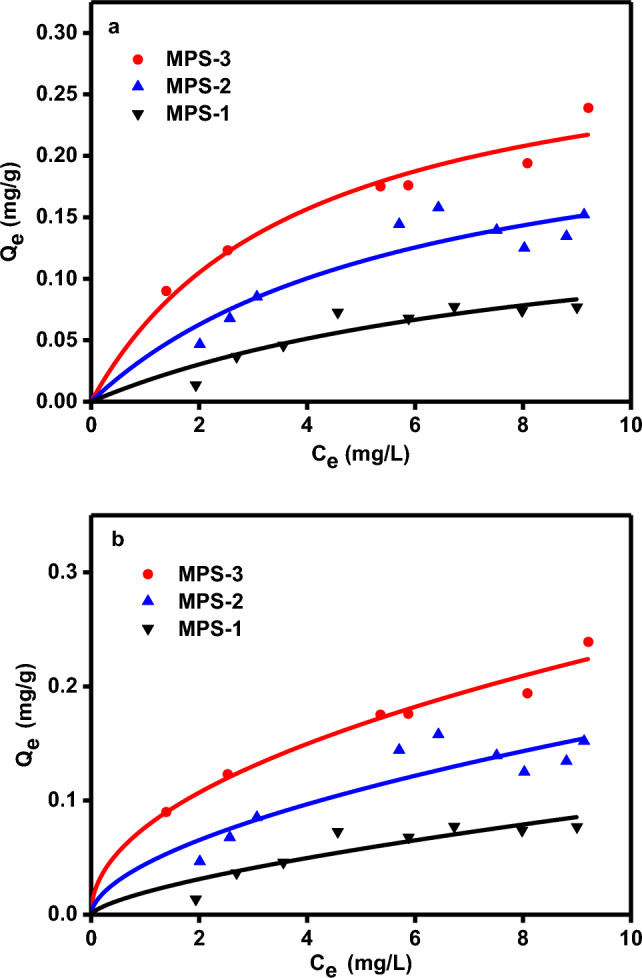
(**a**) Langmuir adsorption isotherm and (**b**) Freundlich adsorption isotherm.

The adsorption of Cu^2+^ on MPS with varying pore sizes (3.2, 3.7, and 4.1 nm) was then investigated under these constant optimal conditions. The adsorption isotherms, depicted in Fig. 3a (Langmuir) and 3b (Freundlich), illustrate the relationship between the equilibrium adsorption capacity (Q_e_) and the equilibrium concentration of Cu^2+^ (C_e_). The data suggest that Cu^2+^ adsorption on MPS is enhanced with increasing pore size, as reflected by the isotherm parameters in Table 2. Specifically, the Freundlich constant (K_f_) and the heterogeneity factor (n) increase with pore size, indicating more favorable adsorption conditions. The Langmuir model, which assumes monolayer adsorption on a surface with uniform energy and no interaction between adsorbed molecules, and the Freundlich model, suitable for multilayer adsorption on heterogeneous surfaces^27^, were applied to the experimental data. The correlation coefficients (R^2^) obtained from these models suggest that the primary mechanism for Cu^2+^ adsorption may involve chemical interactions with the -OH groups on MPS or physical adsorption due to electrostatic forces^28^.

**Table 2:** Parameters of Langmuir and Freundlich model determined by non-linear method.

Sample ID	D_DFT_ (nm)	Exp.Q_e_ (mg/g)	Freundlich Q_e_ = K_f_ C_e_ (1/n)			Langmuir Qe = Q_m_bC_e_/(1 + bC_e_)		
			n	K_f_	R^2^	Q_m_	b	R^2^
MPS-1	3.2	0.09	1.4894	0.0196	0.8683	0.1665	0.1112	0.8939
MPS-2	3.7	0.15	1.7655	0.0441	0.8793	0.2515	0.1659	0.9037
MPS-3	4.1	0.23	2.0654	0.0765	0.9832	0.3090	0.2570	0.9824

**Figure 4:**
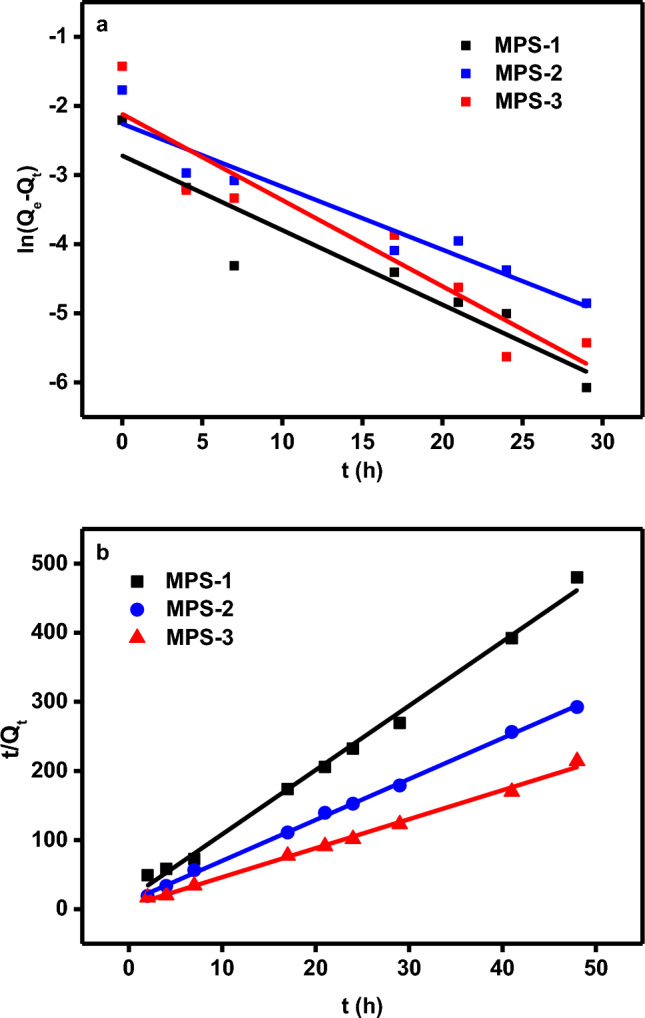
(**a**) Kinetics of Cu^2+^ adsorption according to the pseudo-first-order model (**b**) Kinetics of Cu^2+^ adsorption according to the pseudo-second-order model.

Thermodynamic parameters presented in Table 3 reveal that the adsorption process is spontaneous at 278 and 298 K, as indicated by the negative ΔG^θ^ values. These values decrease with increasing temperature, suggesting enhanced spontaneity at higher temperatures. The positive ΔH^θ^ values for all pore sizes indicate that the adsorption process is endothermic^29^. The positive ΔS^θ^ values suggest increased disorder at the solid–liquid interface during adsorption, consistent with increased adsorption capacity at higher temperatures. Kinetic studies modeled using pseudo-first-order and pseudo-second-order equations are shown in Fig. 4a, b. The pseudo-second-order model, with R^2^ values greater than 0.9937, better describes the experimental data (Table 4), implying that a chemical adsorption process^30^ likely governs the adsorption of Cu^2+^ on MPS. These rate constants decrease with increasing pore size, further supporting this conclusion.

**Table 3:** Parameters of Langmuir model and thermodynamic parameters for the adsorption of Cu^2+^ in MPS at two different temperatures.

Sample ID	D_DFT_ (nm)	T(K)	Langmuir parameters			Thermodynamic parameters		
			Q_m_	b	R^2^	△G (kJ/mol)	△H (kJ/mol)	△S (J/mol)
MPS-1	3.2	278	0.1225	0.1094	0.8862	−20.46	0.67	76
MPS-1	3.2	298	0.1665	0.1112	0.8939	−21.98	0.67	76
MPS-2	3.7	278	0.1755	0.1174	0.9078	−20.63	11.89	117
MPS-2	3.7	298	0.2515	0.1659	0.9037	−22.97	11.89	117
MPS-3	4.1	278	0.2846	0.1664	0.9509	−21.43	14.99	131
MPS-3	4.1	298	0.3090	0.2570	0.9824	−24.05	14.99	131

**Table 4:** Parameters of pseudo first-order and pseudo second-order kinetic model determined by linear method.

SampleID	D_DFT_(nm)	Exp.Q_e_(mg/g)	Pseudo-first-order models ln(Q_e_ – Q_t_) = ln Q_e_ – k_1_t			Pseudo-second-order models t/Q_t_ = (1/k_2_Q_e_^2^) + (1/Q_e_)		
			Q_e.cal_	k_1_	R^2^	Q_e.cal_	k_2_	R^2^
MPS-1	3.2	0.09	0.07	0.1078	0.8558	0.11	5.5468	0.9937
MPS-2	3.7	0.15	0.10	0.0910	0.9031	0.17	3.1885	0.9988
MPS-3	4.1	0.23	0.12	0.8615	0.8615	0.24	3.9902	0.9950

#### The spectroscopic analysis of Cu^2+^ adsorption on MPS

**Figure 5:**
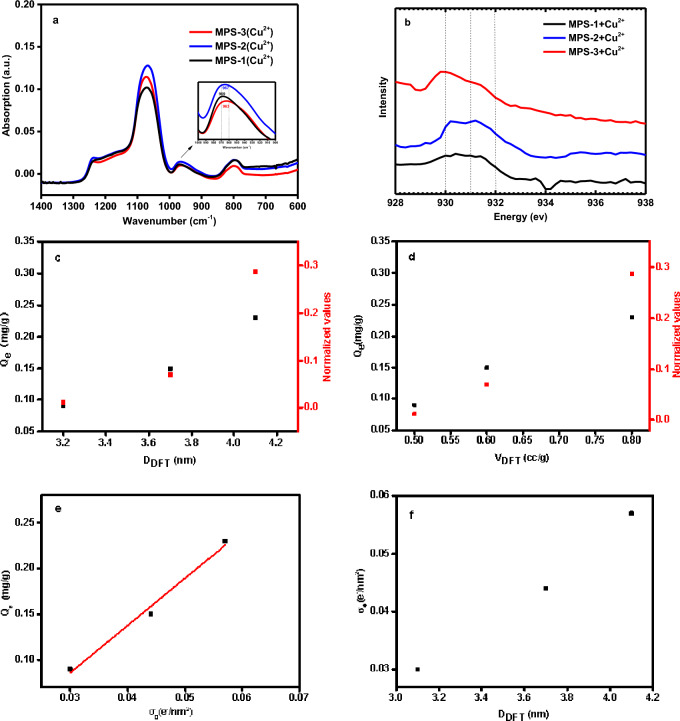
(**a**) ATR FT-IR sorption spectra of Cu^2+^ adsorption on MPS, (**b**) Cu L3-edge XANES spectra of MPS after Cu^2+^ adsorption, (**c**) Changes of relative absorption intensity and Cu^2+^adsorption capacity with surface charge density, (**d**) Changes of Cu^2+^adsorption capacity with pore size, (**e**) Changes of Cu^2+^adsorption capacity with surface charge density and (**f**) Changes of surface charge density with pore size.

In the initial phase of our investigation^31^, comprehensive experiments were conducted to explore the interaction between Cu^2+^ and MPS. Attenuated Total Reflectance Infrared (ATR-IR) analysis provided insights into the chemical changes occurring on the MPS surface before and after Cu^2+^ adsorption. As illustrated in Fig. 5a, the IR spectra of MPS exhibit characteristic peaks at 1082, 970, and 800 cm^−1^. The peaks at 1082 and 800 cm^−1^ correspond to the asymmetric stretching vibrations of Si–O–Si bonds, while the band at 970 cm^−1^ is attributed to the stretching vibration of Si–OH groups. The observed red shift in the stretching modes of Si–O– groups, with features at 969, 967, 965, and 963 cm^−1^, suggests the potential formation of Si–O–Cu bonds^32^, indicating the interaction between Cu^2+^ ions and the MPS surface.

To further investigate these interactions, infrared vibrational spectroscopy experiments were conducted, utilizing the principle of spectral difference to observe changes in the materials^33,34^. The relative content of Cu^2+^ in the samples was quantified by evaluating the integrated intensities of the Si–O– related peaks at 970 cm^−1^, following spectral normalization. The ratio of I_970_/I_800_ was explicitly used to assess the Cu-MPS quantity qualitatively. As depicted in Fig. 5a, the trend of the I_970_/I_800_ ratio correlates with the adsorption capacity, suggesting that larger mesopore sizes (3.1–4.1 nm) enhance the formation of Si–O–Cu bonds, as indicated by the increased IR peak intensities.

Additionally, Cu L_3_ XANES spectroscopy was employed to analyze the electronic structure of Cu-MPS, as shown in Fig. 5b. The spectrum reveals a peak corresponding to the transition from the 2p_3/2_ level to the highest unoccupied 3d state, with the maximum absorption peak observed at approximately 930.1 eV and a shoulder peak at 930.8 eV. These features are attributed to CuO_4_ tetrahedra and CuO_6_ octahedra^35–38^, respectively, suggesting the formation of Si–O–Cu bonds during the adsorption process. The increase in peak intensity at 930.1 and 930.8 eV, along with slight positional shifts, may be attributed to the enhanced adsorption capacity of MPS for Cu^2+^ with increased pore size.

This spectroscopic analysis confirms the interaction between Cu^2+^ ions and MPS, highlighting the role of mesopore size in facilitating the adsorption process. By focusing on nanostructured silica with similar pore structures, our study aims to elucidate the adsorption mechanism of copper ions, providing insights into the performance of MPS in capturing Cu^2+^ from aqueous solutions.

### The analysis of the adsorption mechanism of Cu^2+^ in MPS

This study offers critical insights into MPS’s adsorption capabilities for Cu^2+^ ions by examining the thermodynamic, kinetic, and equilibrium aspects of the process. The findings indicate that the adsorption mechanism likely involves a combination of electrostatic interactions, coordination bonding, and the influence of nano- confinement and charge regulation.

Further investigation into the relationship between surface charge density, mesoporous diameter, and metal ion adsorption, reveals a direct correlation. An increase in surface charge density, as shown in Fig. 5e, and mesoporous diameter, as depicted in Fig. 5f, corresponds to enhanced adsorption of Cu^2+^. The adsorption capacity also increases with pore volume (Fig. 5d), suggesting that the occupancy of Cu^2+^ within mesopores is significant, especially when the ions are not arranged in a monolayer. The quantity of Cu^2+^ adsorbed is intimately linked to the mesoporous surface charge density, a function of both the mesoporous diameter and the specific surface area.

**Figure 6:**
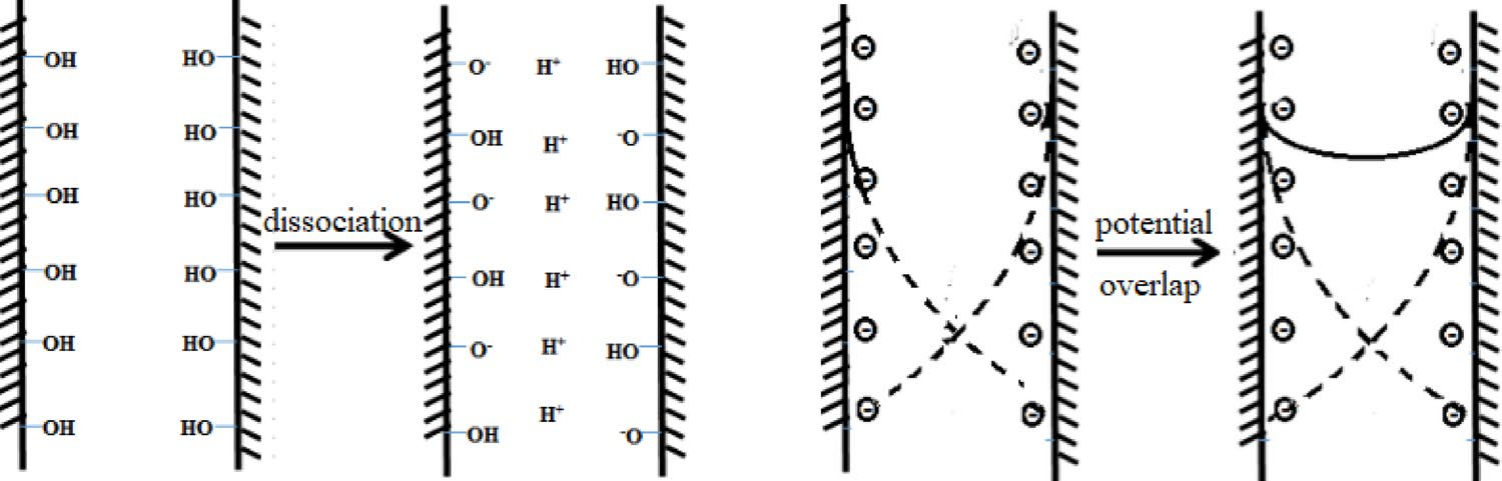
Schematic diagram of potential superposition mechanism.

When mixing MPS with water, the silicon hydroxyl groups dissociate, forming a negatively charged silica surface and a resultant surface potential. In our experiments, using small mesopores resulted in effectively overlapping potentials/double layers within the mesopores, as illustrated in Fig. 6. This overlap impacts the ionization of silicon hydroxyl groups inside the pores. Typically, the pK_a_ value decreases with increasing pore size, which promotes more remarkable dissociation and a higher adsorption capacity for Cu^2+^. This effect is referred to as the potential overlapping mechanism.

The experimental results, as shown in Figure S4, suggest that the increased equilibrium adsorption capacity of copper ions on nanostructured silica with pore sizes below 3.1 nm (0.8 nm) and above 4.1 nm (4.4 nm) can be attributed to differences in their synthesis methods and pore structures. Notably, the 0.8 nm pores are classified as structure-accumulated, whereas the 4.4 nm pores feature numerous micropores on their walls. Consequently, the study concentrates on nanostructured silica with analogous pore structures to illuminate the copper ion adsorption mechanism. By examining nanostructured silica with similar pore configurations, we can better understand its adsorption performance for Cu^2+^.

**Figure 7:**
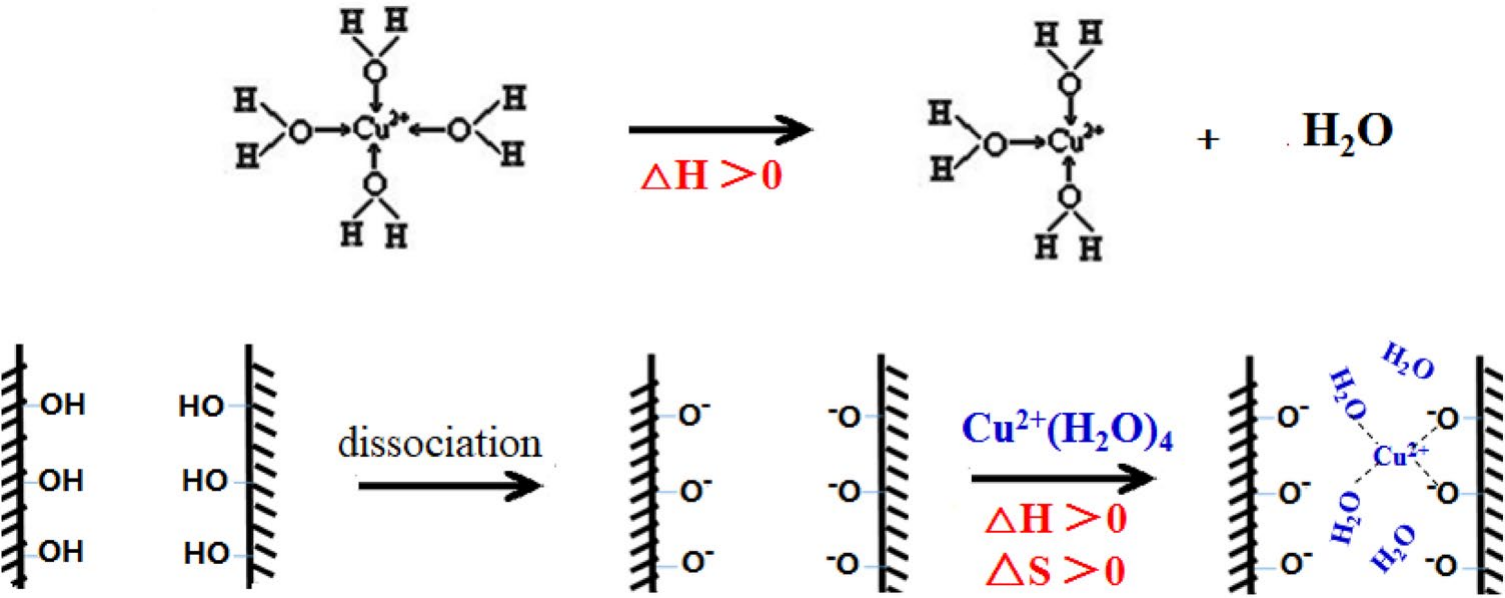
Schematic diagram of energy change in the adsorption process.

During the adsorption process, the desolvation of hydrated Cu^2+^ is an endothermic step, as indicated in Fig. 7. Concurrently, forming new bonds between Cu^2+^ and the adsorbent's surface is an exothermic reaction. Prior thermodynamic analysis, represented in Table 3, suggests that the overall adsorption of Cu^2+^ onto MPS is a spontaneous process characterized by an increase in entropy (ΔS > 0) and endothermic heat absorption (ΔH > 0). With larger pore sizes, the potential overlap in the double layer diminishes, reducing the interaction between the pore wall and Cu^2+^. This also weakens the pore wall’s capacity to bind water molecules and hydrated ions, leading to a rise in the endothermic ΔH and an increase in the system's entropy change ΔS. Thus, the thermodynamic analysis supports the conclusion that adsorption is a spontaneous and favorable process.

## Conclusions

This research explores the thermodynamic and kinetic aspects of Cu^2+^ adsorption on mesoporous silica with different pore sizes, analyzing the adsorption mechanism through surface charge density and spectroscopic methods. The study found that the adsorption aligns closely with the Langmuir model, indicating an endothermic and entropy-increasing spontaneous process with increasing mesopore size. Adsorption kinetics follows a pseudo-second-order model, suggesting a chemical adsorption process supported by spectroscopic evidence showing inner-sphere coordination complexes between Cu^2+^ and mesoporous silica. These findings demonstrate that Cu^2+^ can adhere to mesoporous silica through inner-sphere and outer-sphere complexes, with surface charge density impacting the adsorption capacity of copper ions within specific pore size ranges.

Research results indicate that in the 3.1–4.1 nm pore size range of mesoporous silica, both the pore size and surface charge density play a crucial role in the adsorption of Cu^2+^. Mesoporous materials with larger pore sizes facilitate the diffusion and adsorption of Cu^2+^, while higher surface charge density significantly increases the adsorption capacity of Cu^2+^. Furthermore, it has been shown that as the pore size increases, the surface charge density also increases, and there is an interaction between pore size and surface charge density, both of which collectively impact the adsorption performance of Cu^2+^. Therefore, when designing and optimizing adsorption materials, it is essential to consider a combination of pore size and surface charge density to achieve optimal adsorption efficiency. A thorough understanding of the characteristics of mesoporous silica materials can help develop more efficient adsorption materials, optimize material performance, and expand their application areas, providing more possibilities for environmental pollution control and resource utilization.

## Experiment and methods

### The preparation of mesoprous silica

Mesoporous silica (MPS) was synthesized using a method proposed by Grün et al^39^. The surface-active agents used were long-chain alkyl trimethylammonium bromides (C_n_H_2n+1_N(CH_3_)_3_^+^Br^-^, C_n_TAB) with varying chain lengths of n = 12, 14, and 16. Mesoporous silica specimens with various pore sizes and improved structural order were obtained by modifying the Grün method and then heating for 18 days at 105° C. The three samples, namely MPS-1, MPS-2, and MPS-3, underwent further calcination at a temperature of 550° C. All reagents with a purity of 99% were procured from Shanghai Aladdin Biochemical Technology Co., Ltd., China. Ultrapure water with a resistivity of 18.25 MΩ·cm was obtained using a Millipore water purification system (Molsheim, Alsace, France).

### The characterization methods

Detailed information was obtained on the pore structure, surface properties, and Cu^2+^ interactions of MPS using the methods below.

Automated gas adsorption: The Autosorb-iQ2-MP equipment (Quantachrome, USA) was used to conduct N_2_ adsorption experiments on the samples. During the experiments, N_2_ with a purity of 99.999% and a cross-sectional area of 0.162 nm^2^ was used, and the pressure range (p/p_0_) was 10^−6^ to 0.99. The MPS samples were degassed at 200 °C for 20 h before testing. The BET model was used to calculate the specific surface area and the NLDFT model was applied to determine the pore size and volume of the samples.

Transmission Electron Microscopy (TEM): The pore structure of the samples was examined by means of a FEI Tecnai G2F20 S-TWIN TMP TEM instrument (FEI, USA) at an accelerating voltage of 200 kV.

Thermogravimetric Analysis (TG): The TG analysis was performed on the samples under an Ar atmosphere within a temperature range of 30–1200 °C. The sample weight ranged from 5 to 10 mg, and the heating rate was set between 5 and 10 °C per minute.

Attenuated total reflectance-fourier transform infrared spectroscopy (ATR-FTIR): The ATR-FTIR measurements were done to investigate the interaction between mesoporous silica and Cu^2+^ using a Bruker Vertex 70 setup. A total of 16 scans were performed in a range from 4000 to 400 cm^−1^ at a resolution of 4 cm^−1^.

X-ray absorption near edge structure (XANES) : The Cu L-edge XANES spectroscopy analyses were conducted at the 4B7B beam-line using synchrotron radiation from the Beijing synchrotron radiation facility at the institute of high energy physics of China. The Cu L-edge XANES spectra data were obtained in the total electron yield (TEY) mode, with an energy step of 0.2 eV covering the range from 915 to 960 eV. In order to enhance the signal peak, the test sample was adsorbed using an 80 ppm Cu^2+^ solution at pH 5, resulting in the mesoporous silica sample obtained.

### Evaluation of surface charge density for MPS

The surface charge densities (σ_0_) and zero point charges (PZC) of MPS were determined using an automated potentiometric titrator (Metrohm 905 Titrando, Switzerland).

Preparation of Suspension: Prior to the testing, 0.05 g of MPS was dissolved in 50 mL of deionized water. The mixture was then stored in a closed container for 24 h to reach equilibrium. To remove carbon dioxide, argon gas was bubbled through the suspension for 5 min. The filtered suspension was used as a blank solution, while another portion of the suspension was used as the sample solution.

pH Adjustment: To avoid dissolution of silica, the pH range was set between 1.5 and 8.0. The suspension was initially adjusted to a pH level of 1.5 using 5% nitric acid. Subsequently, a 0.5 mol/L sodium hydroxide solution was dropwise added until the pH value of 8.0 was reached.

Potentiometric Titrator Settings: The titrator was configured with respect to the following parameters: dV (minimum) = 0.0005 mL, dV (maximum) = 0.2 mL, signal drift = 0.1 mV/min, dt = 150 s, minimum time = 150 s, and maximum time = 10,000 s.

### Cu^2+^adsorption experiments on MPS

The Cu^2+^ adsorption experiments were conducted using MPS systems with different pore sizes. The adsorbent was accurately weighed and placed in 25 mL conical flasks. Then, 10 mL of the Cu^2+^ solution with various concentrations was added. The pH of the solutions was adjusted to 5 using 1% HNO_3_. The flasks were kept in a temperature-controlled shaker at 25 °C and agitated for 24 h to reach adsorption equilibrium. The resulting filtrates were obtained by filtering through a 0.45 μm PVDF syringe filter. The concentration of Cu^2+^ in each solution was measured using an atomic absorption spectrophotometer (AAS, 990SUPER, China). Three parallel samples were prepared, and their average values were taken. The adsorption capacity for heavy metal ions was calculated using the equation: Q_e_ = (c_0_−c_e_) × V / m, where Q_e_ is the equilibrium adsorption capacity (mg/g), c_0_ is the initial mass concentration of Cu^2+^ (mg/L), c_e_ is the equilibrium mass concentration of Cu^2+^ (mg/L), V is the volume of the solution (mL), and m is the mass of the adsorbent used (g).

### Supplementary Information

Supplementary Figures.

## Data Availability

Data is provided within the manuscript or supplementary information files.
