# Supplementary material for: Understanding the relationship between pore size, surface charge density, and Cu2+ adsorption in mesoporous silica
Source: Sci Rep. 2024 Jun 12;14:13521. doi: 10.1038/s41598-024-64337-5 (PMC11169565; doi:10.1038/s41598-024-64337-5)
Supplement: Supplementary file 1 — Supplementary Figures. [file 41598_2024_64337_MOESM1_ESM.doc]

**Understanding the relationship between pore** **size, surface charge density, and Cu^2+^ adsorption in mesoporous silica**

Yanhui Niu^1^, WenbinYu^2^, Shuguang Yang^2^, Quan Wan^*,2^

^1^School of Chemistry and Materials Science, Guizhou Education University, Guiyang 550018, China

^2^State Key Laboratory of Ore Deposit Geochemistry, Institute of Geochemistry, Chinese Academy of Sciences, Guiyang 550081, China

*Corresponding author. wanquan@vip.gyig.ac.cn

**Figure S1. Low-angle XRD of MPS**

**Figure S2. (a) Effect of pH on adsorption and (b) Effect of time on adsorption**

**Figure S3. (a) Effect of solid-liquid ratio on adsorption.**

**(b) Uptakes of Cu^2+^ as a function of the used adsorbent dosage.**

**Figure S4 Changes of Cu^2+^adsorption capacity with pore size**
